# Fluoride Retention in Nocturnal Salivary Sediment After Use of a 5000 ppm F Dentifrice: A Randomized Crossover Clinical Trial

**DOI:** 10.1155/tswj/6646481

**Published:** 2025-11-29

**Authors:** Yarlla Franco, Evanildo Paz, Lyzia Rezende, Glauber Vale

**Affiliations:** Restorative Dentistry Department, Federal University of Piauí, Teresina, Piauí, Brazil

**Keywords:** dentifrices, fluoride, saliva

## Abstract

Fluoride (F) is an essential strategy for caries control, with fluoridated dentifrices being the most effective form of its use. Studies have shown that salivary sediment retains more F than the supernatant, contributing to its maintenance in the oral environment. This study was aimed at evaluating the bioavailability of F in salivary sediment after the use of a high-F dentifrice during the nocturnal period. In this in vivo crossover study, 10 participants used a dentifrice containing 5000 ppm F and collected saliva during diurnal and nocturnal periods. These samples were analyzed to compare F concentrations in the salivary supernatant and sediment using an ion-specific electrode. A two-way ANOVA was used for statistical analysis, considering the factors “saliva compartment” and “period,” with a significance level of 5%. No significant differences were observed between the two periods at baseline (*p* > 0.05); however, in both periods, F levels were significantly higher in the sediment than in the supernatant (*p* < 0.05). Immediately after brushing, a peak in F concentration was observed in all compartments, followed by a gradual decline over time. After 2 h, F retention remained consistently higher in the sediment compared to the supernatant, especially during the nocturnal period (*p* < 0.05). The area under the curve (AUC) confirmed higher F bioavailability at night, with values (mean ± SD, *μ*g F/mL.min^−1^) of 3590.1 ± 450.0 for nocturnal sediment, 2598.2 ± 209.9 for diurnal sediment, 2526.7 ± 579.2 for nocturnal supernatant, and 2078.4 ± 442.8 for diurnal supernatant (*p* = 0.03). These findings demonstrate that bedtime brushing with a 5000-ppm F dentifrice effectively prolongs F retention in the oral environment, especially in the salivary sediment.

**Trial Registration:**

Brazilian Clinical Trials Registry (REBEC) identifier: RBR-10bhvcr4

## 1. Introduction

The use of fluoride is widely recognized as a key strategy for controlling dental caries at the community, professional, and individual levels [1]. Its primary mechanism of action is topical, promoting the remineralization of early carious lesions and reducing the demineralization of healthy dental tissues [2]. Among the various methods of fluoride use, fluoridated dentifrices are acknowledged as the main factor contributing to the decline in caries prevalence in recent decades. They are considered the most rational approach, as they combine mechanical biofilm disruption with fluoride's chemical action on demineralization and remineralization processes [3].

High-fluoride dentifrices were developed to increase the concentration of fluoride in the oral microenvironment compared to conventional formulations and are recommended for individuals at high risk of caries [4]. Their use has proven effective in preventing both mineral loss and the progression of dentin lesions in vitro [5]. However, for fluoride to exert its protective effects, it must be present in a free or soluble form within the aqueous oral environment, such as in biofilm fluid and/or saliva [6].

Human saliva can be separated by centrifugation into supernatant saliva, which is cell-free, and salivary sediment, which contains most of the oral microbiome phylotypes, cellular components, proteins, and food debris [7, 8]. It is known that after brushing with fluoridated dentifrices, variations in fluoride bioavailability occur across different salivary compartments: the supernatant, the sediment, and the biofilm, largely depending on the formulation used [9]. Several studies have shown that fluoride concentration in the salivary supernatant is significantly lower than in the sediment [5].

Following the use of fluoridated dentifrices, fluoride bioavailability in saliva rises sharply for a short period before gradually declining. Therefore, maintaining elevated salivary fluoride levels throughout the day and night is particularly important for high-risk groups, such as individuals with active carious lesions, patients undergoing orthodontic treatment, and elderly individuals, especially those with salivary gland hypofunction [10]. Although the benefits of high-fluoride dentifrice are well documented, limited data exist on their effectiveness during nighttime, when salivary flow is reduced. Hence, the objective of the present study is to evaluate the bioavailability of fluoride in salivary sediment following the use of high-fluoride dentifrices during this period.

## 2. Materials and Methods

### 2.1. Ethical Aspects

This study was approved by the Research Ethics Committee of the Federal University of Piauí (CEP/UFPI) under Opinion Number 483.913. All participants signed an informed consent form, and the study was conducted in accordance with Resolution No. 466/2012 of the National Health Council (Ministry of Health, Brasília, DF) and the Declaration of Helsinki. The study followed the CONSORT (Consolidated Standards of Reporting Trials) guidelines, and no deviations or amendments occurred between the registered protocol and the final manuscript.

### 2.2. Experimental Design

**Figure 1:**
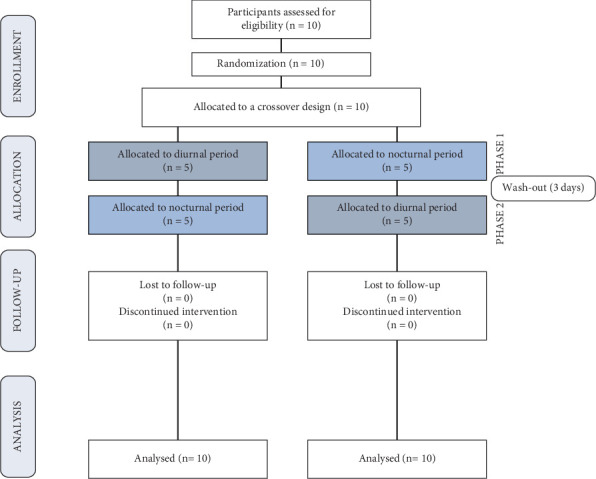
Flowchart of the study.

A randomized, short-term clinical trial with a crossover design was conducted involving 10 adults of both sexes. The sample size was based on prior studies [4, 11] that showed adequate statistical power (≥ 80%) for intraindividual comparisons. During two experimental phases, participants randomly performed a single brushing with a commercial high-fluoride dentifrice (5000 ppm F) during either the diurnal or nocturnal period. Before the experimental protocol (lead-in period) and between the phases (wash-out periods), participants used dentifrices without F for 3 days. Randomization of diurnal/nocturnal brushing order was performed via https://random.org/ by an investigator not involved in data collection. The experimental flowchart is shown in Figure 1.

### 2.3. Saliva Sample Collection

Each participant received a kit containing a toothbrush, saliva collection tubes, a standardized disposable cup for postrushing rinsing (50 mL), a fluoride-free dentifrice (placebo), and the designated dentifrice for the study phase. All dentifrices, except the placebo, were packaged in identical tubes to ensure blind identification. Before starting the saliva collections, participants received detailed verbal and written instructions: (1) All participants were instructed to begin the experiment by using the placebo dentifrice on the first day. (2) On the following day, they brushed with the designated fluoride dentifrice. (3) They were instructed to brush their teeth for 1 min, following previous fluoride bioavailability studies [4, 11], to ensure standardization and reproducibility, using an amount of dentifrice equivalent to the length of the toothbrush bristles, approximately 1 g. This weight was predetermined in the laboratory using analytical scales by applying dentifrice along the transverse length of the brush head. (4) After spitting out the foam, a 50-mL tap water rinse (measured with the cup provided in the kit) was used for 10 s to simulate real-world oral hygiene behavior [7].

**Figure 2:**
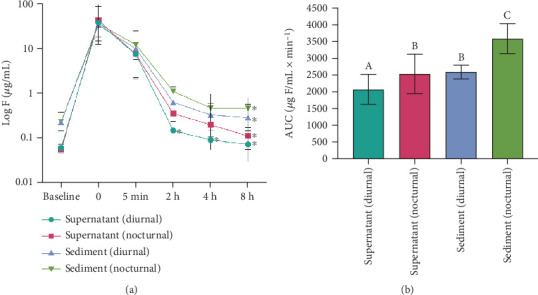
(a) Kinetics (*m*ean ± SD, *n* = 10) of F concentration in supernatant and salivary sediment (micrograms per milliliter) according to time after brushing in the diurnal or nocturnal period. Asterisks indicate the time at which F concentration is similar to baseline by Dunnett's test (*p* < 0.05). Time points on the *x*-axis are displayed at equal intervals to facilitate visualization. (b) Mean AUC of F concentration in supernatant and salivary sediment as a function of time (*μ*g F/mL.min^−1^) according to salivary compartment and period (*n* = 10). Vertical bars indicate standard deviation, and distinct letters indicate statistical difference (*p* < 0.05).

Considering the crossover design, no standardized brushing technique was required. Unstimulated saliva samples were collected in both day and night periods with a 3-day washout at the following time points: before brushing (baseline); immediately after (Time 0); and at 5 min, 2 h, 4 h, and 8 h postbrushing. Participants collected approximately 3 mL of saliva in prelabeled tubes provided in the kit. For nocturnal collections, participants were awake during the 0- and 5-min collections and went to sleep immediately afterward. At 2 and 4 h, participants woke up briefly, collected the saliva, and returned to bed. Samples were placed in hermetically sealed bags and stored in the freezer (Figure 2).

### 2.4. Determination of Fluoride Concentration in Saliva

Fluoride concentration in the samples was measured using a fluoride ion-specific electrode (Analyser 18AF, São Paulo, Brazil) connected to an ion analyzer (Orion EA-740). From each tube, 1 mL of saliva was transferred to an Eppendorf tube and centrifuged at 5000 g for 3 min. Then, 0.5 mL of the salivary supernatant was mixed with an equal volume of TISAB II buffer (composition in Table 1). The remaining supernatant was discarded, leaving the sediment at the bottom of the tube. To this sediment, 0.5 mL of TISAB II was added and vortexed for 15 s [11]. Fluoride concentrations in the saliva compartments were calculated by linear regression from a calibration curve prepared with standards ranging from 0.125 to 32 *μ*g F/mL under the same conditions as the test samples.

### 2.5. Statistical Analysis

A two-way analysis of variance (ANOVA) was used as the statistical model, considering the factors “period” (day vs. night) and “saliva compartment” (supernatant vs. sediment). The Shapiro–Wilk test confirmed normal data distribution after log_10_ transformation. Data were analyzed using two-way ANOVA followed by Tukey's post hoc test. Fluoride levels at each time point were compared to baseline values using ANOVA with Dunnett's multiple comparison posttest. All statistical analyses were performed using GraphPad Prism Version 10.05 for Windows, with the level of significance set at 5%.

## 3. Results

Table 1 presents the fluoride concentrations (micrograms of fluoride per milliliter) in the salivary supernatant and sediment following the use of a high-fluoride dentifrice during both diurnal and nocturnal observation periods. At baseline (before brushing), no significant differences were observed between day and night for either the supernatant or sediment (*p* > 0.05). However, in both periods, fluoride levels were significantly higher in the sediment than in the supernatant (*p* < 0.05). Immediately after brushing (Time 0), a peak in fluoride concentration was observed across all salivary compartments and time points analyzed. Five minutes postbrushing, fluoride levels decreased but remained relatively elevated, with no statistically significant differences between groups (*p* > 0.05). Over time, fluoride bioavailability declined rapidly within the first 2 h of postbrushing. The salivary sediment consistently retained higher fluoride levels than the supernatant from 2 h onward (*p* < 0.05), particularly during the nocturnal period, supporting the hypothesis that sediment serves as a fluoride reservoir.

Figure 2a illustrates the kinetics of fluoride concentrations in both the salivary supernatant and sediment during the diurnal and nocturnal periods following brushing with a high-fluoride dentifrice. A sharp peak is evident immediately after brushing (Time 0), followed by a gradual decline in fluoride levels over time. After 8 h, fluoride levels approached baseline values for all groups evaluated, except for supernatant in the diurnal period, which reached baseline values of fluoride after 2 h (*p* < 0.05).  Figure 2b presents the area under the curve (AUC) for fluoride concentrations in both salivary compartments, revealing the overall bioavailable fluoride across the two periods. The nocturnal period exhibited greater fluoride retention, with higher AUC values in both supernatant and sediment compartments. This effect was particularly pronounced in the sediment, which demonstrated significantly greater fluoride retention (*p* < 0.05).

Table 2 shows the ratio of mean fluoride concentrations in the sediment relative to the supernatant during each observation period. At baseline, fluoride bioavailability was higher in the sediment during both periods. However, immediately after brushing, fluoride concentrations were higher in the supernatant. At 5 min postbrushing, fluoride levels remained higher in the supernatant during the nocturnal period, while the diurnal period showed no substantial change from the previous time point. Interestingly, the highest fluoride concentration in the sediment was observed 2 h postbrushing during the diurnal period, indicating a shift of fluoride from the liquid to the solid phase. Notably, at 8 h postbrushing during the nocturnal period, there was an increase in the fluoride ratio, suggesting that fluoride gradually accumulates in the sediment. These findings indicate that although the initial fluoride peak occurs in the supernatant, a subsequent shift occurs, returning fluoride to the sediment, which acts as a reservoir capable of storing and gradually releasing fluoride over time.

## 4. Discussion

This study isolated the supernatant and sediment fractions of whole saliva to evaluate how the use of a high-fluoride dentifrice influences fluoride kinetics in these compartments. Assessing this parameter is especially relevant for the development of new fluoride-based products or preventive therapies with varying concentrations [11]. Fluoride affects salivary levels not only in the liquid phase (supernatant) but also significantly in the solid fraction (sediment), which functions as a biological reservoir. The sediment is capable of storing fluoride and gradually releasing it into the supernatant, thereby maintaining its availability in the soluble phase, where it plays a central role in the demineralization and remineralization processes [6].

Our results showed increased fluoride concentrations in both salivary compartments during the diurnal and nocturnal periods, consistent with studies demonstrating that the concentration and amount of fluoride in dentifrice influence its bioavailability [11–13]. No significant differences were observed between the two periods at baseline; however, within each period, the sediment consistently exhibited higher fluoride levels, confirming that this compartment retains fluoride more efficiently than the supernatant [4, 8]. Even without additional fluoride exposure, higher fluoride levels in salivary sediment hours after brushing suggest that it serves as a stable and slowly releasing reservoir.

Immediately after brushing with a 5000 ppm F dentifrice, fluoride concentration peaked across all compartments, as expected for higher concentration formulations [14]. The rapid decline within the first few minutes represents the initial clearance phase, followed by a slower reduction associated with fluoride's interaction with organic and inorganic sediment components. Indeed, after toothbrushing, salivary fluoride availability can rise more than 100-fold within the first few minutes but usually returns to baseline within about 2 h [8, 13]. This clearance pattern is influenced by factors such as the chemical form and solubility of the formulation, rinsing behavior, and salivary flow rate [7, 8]. From 2 h onward, fluoride distribution shifted toward the sediment, indicating its capacity to retain and gradually release fluoride over time [4].

The higher AUC values observed during the nocturnal period, especially in the sediment, demonstrate greater fluoride availability when salivary flow is physiologically reduced [15]. Bedtime brushing is therefore a critical opportunity for prolonging fluoride exposure [16], particularly for individuals with hyposalivation, high caries activity, or fixed orthodontic appliances. The sediment's ability to slowly release fluoride provides extended protection during physiological nocturnal hyposalivation [17, 18]. The clinical implications of these findings support the recommendation of brushing with a high-fluoride dentifrice before bedtime. This practice may help prevent demineralization resulting from the absence of protective salivary effects [19] and the associated increase in oral acidity. Moreover, the study highlights a direct relationship between the fluoride concentration in dentifrice and the level of fluoride retained in saliva, emphasizing the efficacy of high-concentration formulations in prolonging fluoride ion bioavailability.

It is plausible that fluoride binds to salivary sediment through ionic or hydrogen bonds, potentially forming CaF_2_-like globules, as previously suggested [4, 6]. Clinically, these findings reinforce the recommendation of bedtime brushing with high-fluoride dentifrice for patients with high caries risk, orthodontic appliances, or reduced salivary flow. Future investigations could further elucidate the chemical nature of fluoride binding within salivary sediment. Some limitations of this study should be acknowledged, including interindividual differences in brushing technique, removal of dentifrice foam, and postbrushing rinsing habits, which may have introduced variability in salivary fluoride levels despite standardized instructions and the crossover design. These aspects should be considered when interpreting the findings.

## 5. Conclusion

Based on the results of this study, it can be concluded that salivary sediment retains fluoride more effectively than the salivary supernatant, particularly during the nocturnal period. Therefore, brushing at night with a high-fluoride dentifrice is effective in maintaining fluoride availability for extended periods. The salivary sediment functions as a fluoride reservoir, which is especially important during sleep, when salivary flow is naturally reduced due to physiological hyposalivation.

## Figures and Tables

**Table 1:** Mean ± SD (*n* = 10) of fluoride concentration in the supernatant or salivary sediment (micrograms of fluoride per milliliter) according to the period evaluated (diurnal or nocturnal).

**Time**	**Diurnal**		**Nocturnal**	
	**Supernatant**	**Sediment**	**Supernatant**	**Sediment**
Baseline	0.06 (0.01) aA	0.23 (0.06) bA	0.06 (0.01) aA	0.26 (0.12) bA
0	55.05 (52.07) aA	49.83 (50.88) aA	60.66 (62.98) aA	48.20 (41.80) aA
5 min	14.14 (18.38) aA	12.72 (7.32) aA	15.72 (24.09) aA	16.47 (16.80) aA
2 h	0.16 (0.08) aA	0.84 (0.70) bA	0.53 (0.58) aB	1.57 (1.49) bB
4 h	0.10 (0.07) aA	0.39 (0.23) bA	0.24 (0.15) aB	0.59 (0.40) bB
8 h	0.12 (0.18) aA	0.35 (0.24) bA	0.14 (0.12) aA	0.52 (0.28) bB

*Note:* Lowercase letters denote significant differences between supernatant and sediment within the same period, and uppercase letters denote significant differences between periods within each salivary compartment (supernatant or sediment), *p* < 0.05.

**Table 2:** Proportion of mean values of fluoride concentration in the sediment to mean fluoride values in the salivary supernatant in the evaluated periods.

**Period**	**Time**					
	**Baseline**	**0**	**5 min**	**2 h**	**4 h**	**8 h**
Diurnal	**3.82**	0.91	0.90	**5.15**	**3.72**	**2.96**
Nocturnal	**4.64**	0.79	**1.05**	**2.98**	**2.50**	**3.68**

*Note:* Bold values indicate higher fluoride concentration in the sediment compared to the supernatant.

## Data Availability

The data that support the findings of this study are available from the corresponding author upon reasonable request.
